# A new way to build cell lineages

**DOI:** 10.7554/eLife.25654

**Published:** 2017-03-23

**Authors:** Xiuwei Zhang, Nir Yosef

**Affiliations:** 1 Department of Electrical Engineering and Computer Science and the Center for Computational Biology, University of California, Berkeley, United States; 1 Department of Electrical Engineering and Computer Science and the Center for Computational Biology, University of California, Berkeley, United Statesniryosef@berkeley.edu; 2 Ragon Institute of Massachusetts General Hospital, MIT and Harvard, Cambridge, United States; 2 Ragon Institute of Massachusetts General Hospital, MIT and Harvard, Cambridge, United States

**Keywords:** germ layer differentiation, context dependence, single-cell RNA-seq, systems biology, Transcriptomics, Human, Mouse

## Abstract

A combination of single-cell techniques and computational analysis enables the simultaneous discovery of cell states, lineage relationships and the genes that control developmental decisions.

**Related research articles** Jang S, Choubey S, Furchtgott L, Zou LN, Doyle A, Menon V, Loew EB, Krostag AR, Martinez RA, Madisen L, Levi BP, Ramanathan S. 2017. Dynamics of embryonic stem cell differentiation inferred from single-cell transcriptomics show a series of transitions through discrete cell states. *eLife* **6**:e20487. doi: 10.7554/eLife.20487

Furchtgott LA, Melton S, Menon V, Ramanathan S. 2017. Discovering sparse transcription factor codes for cell states and state transitions during development. *eLife* **6**:e20488. doi: 10.7554/eLife.20488

The identity or state of a cell depends on numerous factors. Some of these factors are transient in nature (such as the stage the cell is at in the cell cycle), while others reflect long-lasting commitments, such as those that occur during the development of stem cells (Novershtern et al., 2011, Graf and Enver, 2009). By making the entire transcriptome available, single-cell RNA sequencing is now allowing researchers to systematically investigate these factors (Wagner et al., 2016; Tanay and Regev, 2017). Specifically, single-cell technology opens the way for developmental biologists who work on the transitions between different cell states to explore three outstanding questions: (1) What are the cell states (both transitional and long lasting or terminal) that comprise a developmental process of interest? (2) What transitions take place between these states? (3) How are these transitions regulated?

Now, in a pair of papers in eLife, researchers at Harvard University and the Allen Institute for Brain Science report a framework that uses whole-genome mRNA expression profiling to address these questions, which they then apply to stem cell differentiation in mouse embryos (Furchtgott et al., 2017; Jang et al., 2017). The basic concept that underlies these two papers concerns the second question, which is about transitions between cell states that have already been defined in advance. Previous attempts to address this question mostly relied on the notion that two cell states are 'close' to each other in their lineage tree if their gene expression profiles are similar (Qiu et al., 2011; Shin et al., 2015). In the first of the papers Leon Furchtgott, Samuel Melton, Vilas Menon and Sharad Ramanathan present an alternative strategy, which was motivated by an investigation of gene expression in B- and T-cells as they developed (Furchtgott et al., 2017).

Combining this gene expression data with what was already known about the lineage relationship between the different states of the B- and T-cells, Furchtgott et al. identified triplets of cell states that exhibited a consistent pattern. Each triplet contained a precursor state and two descendant states, and for many transcription factor genes, the expression in one member of the triplet was much less than in the other two members. Furthermore, the member of the triplet with low levels of gene expression was rarely the 'central' state, which can represent either a common precursor for the two other states, or a transitional state between them (see Figure 1). This finding is consistent with previous work which showed that cell differentiation involves the selective silencing of certain transcription factors (Graf and Enver, 2009; Novershtern et al., 2011), or that transcriptional profiles often exhibit a 'single-pulse' pattern during development (Yosef and Regev, 2011).

**Figure 1.**
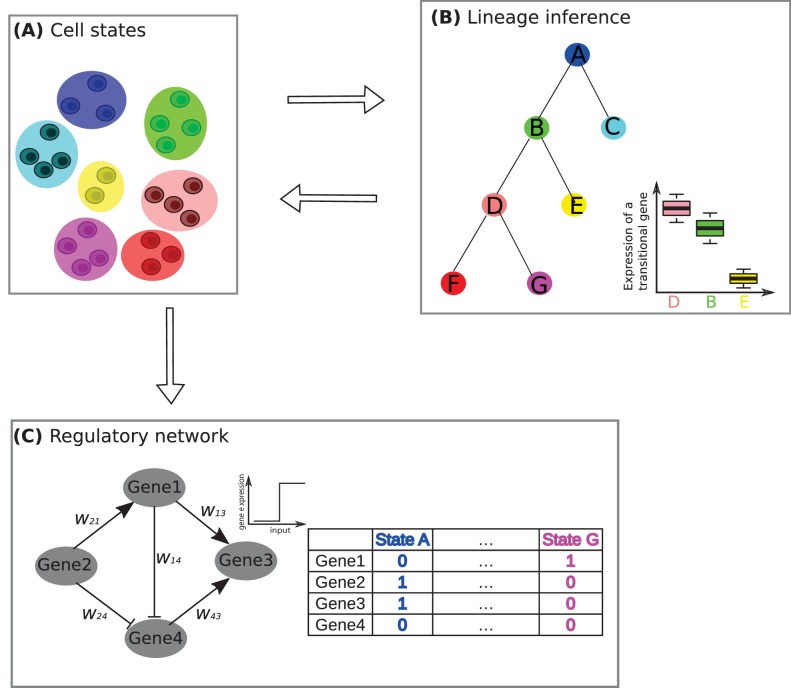
A framework for studying developmental processes with single-cell RNA sequencing. (**A**) The first challenge is to identify the different cell states. Jang et al. used single-cell RNA sequencing and other techniques to identify nine different cell states, based on them having similar mRNA profiles, during the early stages of development in a mouse embryo. Here, for the purposes of illustration, we show a system in which there are seven cell states (denoted by A–G), with two, three or four cells in each state. (**B**) The second challenge is to determine how these states fit into a lineage tree. This process is helped by the fact that the states form triplets (such as D-B-E or B-D-F, where the central state is B and D respectively), with one non-central member of the triplet having low levels of expression for certain 'transitional' transcription factor genes (see boxplot, where E has low levels of gene expression, whereas B and D have high levels). Furchtgott et al. couple these two challenges by an iterative process of first inferring cell sub-populations, then identifying a lineage tree over these sub-populations, and then restarting the process, this time using only the transitional genes to define the cell sub-populations. (**C**) The third challenge is to understand how transcriptional regulation controls cell development in this system. In the example shown here it is assumed that a network of four transcription factor genes (or clusters of co-regulated genes) are involved in regulation. By comparing many possible networks that can be formed by four genes (or clusters of genes) and have seven steady states (one for each of the cell states identified in A), it is possible to make predictions of the interaction between pairs of transcription factors. In this example the state A corresponds to genes 2 and 3 being expressed (1) and genes 1 and 4 not being expressed (0), while state G corresponds to gene 1 being expressed and genes 2, 3 and 4 not being expressed. The expression of a gene is determined by summing over the influences of its expressed neighbors: for example, under some parametrization, gene 3 in this network will be determined as expressed if genes 1 and 4 are on.

Furchtgott et al. then developed a statistical method to test whether a given triplet of states reflects a true developmental progression and, if so, in what order. The method is based on identifying 'transitional' genes that are clearly expressed at low levels in one of the states, and testing whether the overall pattern of transitions (while looking at the entire set of transcription factor genes) is likely due to the presence of a lineage relationship. All the triplets that 'pass' this test are then merged into one global lineage tree. This new strategy opened up the possibility of categorizing all the cells in a single-cell RNA sequencing dataset into developmental states, without knowing in advance what these states were. To achieve this, which essentially involves answering the first of our three questions, Furchtgott et al. developed an iterative algorithm that seeks to partition the cells into clusters (each representing a separate state), such that the overall likelihood of lineage relationships between these clusters is high.

Ramanathan, Sumin Jang and co-workers – including Jang and Sandeep Choubey as joint first authors – then used this approach to do two things: first, they identified nine cell states that occur as embryonic stem cells undergo differentiation and eventually become progenitor cells for the different germ layers in a mouse embryo; second, they organized these nine states into a lineage tree (Jang et al., 2017). The next challenge was to find out how transcriptional regulation controlled cell development in this system.

In the past researchers have focused primarily on the transitions between states (see, for example, Novershtern et al., 2011; Shin et al., 2015). However, when Jang et al. used flow cytometry and live-cell microscopy to monitor various biomarkers for the cell state, they found that the different cell states were relatively stable, while the transitions between them occurred more rapidly. This prompted them to focus on the states themselves, rather than the transitions between them. In particular they looked for a network of interactions between sets of transcription factors that could converge to a number of different steady states (Huang et al., 2009), with each of these steady states representing a particular cell state.

Since the number of networks that exhibit this property is extremely high, Jang et al. were not able to explore all of them. Rather, they explored a sample, looking for relationships between transcription factors that were consistent across many networks. This allowed them to make predictions about the relationships between various transcription factors, and how these relationships depended on the cell state. For example, they predicted (and then experimentally verified) that the expression of Oct4 is more sensitive to the over-expression of Sox2 when a cell is in an epiblast-like state than when it is in a state that is like an embryonic stem cell.

While new tools, particularly single-cell RNA-sequencing, are proving to be highly productive, we could learn much more by measuring other molecular profiles within the cells. For instance, knowing more about the chromatin state of single cells could help a lot when categorizing them into cell states and trying to identify the most active transcription factors (Wagner et al., 2016; Tanay and Regev, 2017). Technologies for lineage tracking can further provide direct observations of cell state transitions (Woodworth et al., 2017). Looking to the future, the ability to collect multiple types of data from single cells, combined with the ability to integrate and interpret all these data in an informative manner, is sure to lead to new insights into how cells change and fate decisions are made during development.
